# The Anti-Inflammatory Effect of a γ-Lactone Isolated from Ostrich Oil of *Struthio camelus* (Ratite) and Its Formulated Nano-Emulsion in Formalin-Induced Paw Edema

**DOI:** 10.3390/molecules26123701

**Published:** 2021-06-17

**Authors:** Salah E. M. Eltom, Ahmed A. H. Abdellatif, Hamzah Maswadeh, Mohsen S. Al-Omar, Atef A. Abdel-Hafez, Hamdoon A. Mohammed, Eiman ME. Agabein, Ibrahim Alqasoomi, Salem A. Alrashidi, Mohammed S. M. Sajid, Mugahid A. Mobark

**Affiliations:** 1 Department of Medicinal Chemistry and Pharmacognosy, College of Pharmacy, Qassim University, Buraydah 51452, Saudi Arabia; m.omar@qu.edu.sa (M.S.A.-O.); a.abdelgalil@qu.edu.sa (A.A.A.-H.); salem11098@gmail.com (S.A.A.); 2 Department of Pharmaceutics, College of Pharmacy, Qassim University, Buraydah 51452, Saudi Arabia; A.Abdellatif@qu.edu.sa (A.A.H.A.); msodh@qu.edu.sa (H.M.); imq3911@gmail.com (I.A.); 3 Department of Pharmaceutics and Industrial Pharmacy, Faculty of Pharmacy, Al-Azhar University, Assiut 71524, Egypt; 4 Department of Pharmacognosy, Faculty of Pharmacy, Al-Azhar University, Cairo 11371, Egypt; 5 Department of Histology, College of Medicine, Qassim University, Buraydah 51452, Saudi Arabia; eimanagabein@qumed.edu.sa; 6 Department of Pharmacology and Toxicology, College of Pharmacy, Qassim University, Qassim 51452, Saudi Arabia; su.mohammed@qu.edu.sa; 7 Department of Pharmacy Practice, College of Pharmacy, Qassim University, Buraydah 51452, Saudi Arabia; mu.mohammed@qu.edu.sa; 8 Department of Pathology, Faculty of Medicine and Health Sciences, University of Kordofan, El-Obied 157, Sudan

**Keywords:** Diclofenac, γ-lactone, nano-emulsion, methylcellulose, Ostrich oil, *Struthio camelus*

## Abstract

The ostrich oil of *Struthio camelus* (Ratite) found uses in folk medicine as an anti-inflammatory in eczema and contact dermatitis. The anti-inflammatory effect of a γ-lactone (5-hexyl-3H-furan-2-one) isolated from ostrich oil and its formulated nano-emulsion in formalin-induced paw edema was investigated in this study. Ostrich oil was saponified using a standard procedure; the aqueous residue was fractionated, purified, and characterized as γ-lactone (5-hexyl-3H-furan-2-one) through the interpretation of IR, NMR, and MS analyses. The γ-lactone was formulated as nano-emulsion using methylcellulose (MC) for oral solubilized form. The γ-lactone methylcellulose nanoparticles (γ-lactone-MC-NPs) were characterized for their size, shape, and encapsulation efficiency with a uniform size of 300 nm and 59.9% drug content. The γ-lactone was applied topically, while the formulated nanoparticles (NPs) were administered orally to rats. A non-steroidal anti-inflammatory drug (diclofenac gel) was used as a reference drug for topical use and ibuprofen suspension for oral administration. Edema was measured using the plethysmograph method. Both γ-lactone and γ-lactone-MC-NPs showed reduction of formalin-induced paw edema in rats and proved to be better than the reference drugs; diclofenac gel and ibuprofen emulsion. Histological examination of the skin tissue revealed increased skin thickness with subepidermal edema and mixed inflammatory cellular infiltration, which were significantly reduced by the γ-lactone compared to the positive control (*p*-value = 0.00013). Diuretic and toxicity studies of oral γ-lactone-MC-NPs were performed. No diuretic activity was observed. However, lethargy, drowsiness, and refusal to feeding observed may limit its oral administration.

## 1. Introduction

Natural products play a prominent role in the treatment of diseases and are of major concern in drug discovery [1,2,3]. Plants are the main renewable source of natural products; however, some other renewable sources such as animals and microorganisms can be used for natural product production [4,5]. Ostrich, *Struthio camelus* is a large flightless bird which belongs to the Ratite family. This family includes three types of birds, ostrich, emu, and rhea, the ostrich lives in African countries, mainly in the areas of rich savanna [6]. Ostrich oil has been used in folk medicine as topical treatment of eczema, psoriasis, dry skin, contact dermatitis, burns, hair growth, dry hair, bedsores, and muscular pain [7]. Emu oil, having similar composition as ostrich oil, has anti-inflammatory and skin desensitizing properties [4]. Topical application of emu oil was shown to reduce inflammation associated with reduced levels of interleukin 1-alpha, tumor necrosis factor-alpha, and other proinflammatory cytokines in a croton-oil-induced inflammation mouse model [4,8,9]; however, there is limited scientific research data on these properties with ostrich oil. The fatty acids’ profile showed that the highest ratios include oleic acid 31.04% followed by palmitic acid 19.26%, then arachidonic acid 15.92%, and erucic acid 6.75% [10].

Although ostrich oil has similarities in chemical composition but not identical to emu oil which showed anti-inflammatory properties [7], it is expected that ostrich oil will have anti-inflammatory activity as emu oil [4]. As ostrich oil is almost triglyceride lipids and is free of phospholipids, consequently it could penetrate human skin. The triglyceride exists in the saponifiable fraction is responsible for the anti-inflammatory activity [11], eventually our search for the part of the saponifiable faction of ostrich oil was focused on, which might be responsible for the anti-inflammatory effect. Inflammation is an immediate and severe response by living tissue to injuries. The primary indicators for inflammations are four; pain, redness, warmness and swelling which is followed by raised blood circulation towards the inflamed area (redness).

The poor solubility of γ-lactone restrained its applications. As a good protection and oral delivery system, an optimal nano-emulsion can be developed by using low-energy emulsification method. These can increase the systematic bioavailability and biological activity. Also, the solubilized γ-lactone in nano-emulsion can be used as an oral delivery system dramatically with improved stability and solubility [12]. Nano-emulsion has been identified as an excellent delivery system for drugs. Nano-emulsion is a heterogeneous system composed of one immiscible liquid dispersed as droplets within a dispersion medium. Nano-emulsions consist of fine oil-in-water dispersions, the size range of covering droplets is 100–600 nm. Nano-emulsions droplets, usually spherical in shape of dispersed particles used for biopharmaceutical assistance [13]. The nano-emulsion drug-delivery system has an adequate storage capacity and thermodynamic stability for drug delivery. The nano-emulsion can contain large quantities of hydrophobic dissolved with their mutual compatibility and ability to get the drugs protected from hydrolysis and enzymatic degradation, making them ideal for parenteral transport [12,14]. There are a few factors that must be considered during the preparation of nano-emulsion, including: the careful choice of surfactant to achieve an ultralow interfacial tension, which is a crucial requirement to produce nano-emulsion. Also, concentration of the surfactant must be high enough to stabilize the microdroplets to create nano-emulsion. The surfactant must be flexible or fluid sufficient to promote the formation of nano-emulsion. Several nano-emulsion evaluation parameters must be kept in mind, such as, one droplet size analysis measured by a diffusion method using a light scattering and particle size-analyzer counter [15]. This study evaluated the potential anti-inflammatory effect of the γ-lactone isolated from ostrich oil and its nano-emulsion against formalin induced edema using diclofenac gel for topical application and ibuprofen for oral administration as standard drugs. Spectrophotometric analysis for both γ-lactone and its nano-emulsion together with the toxicity and diuretic activity of the oral γ-lactone-MC-NPs were studied. Also, skin tissues from rats treated with topical γ-lactone were histologically examined.

## 2. Results and Discussion

### 2.1. Isolation and Characterization of γ-Lactone (5-hexyl-3H-furan-2one)

**Figure 1:**
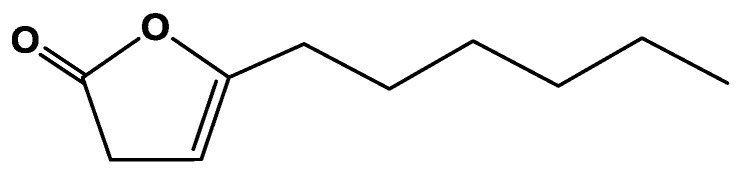
Chemical structure of 5-hexyl-3H-furan-2-one.

The purified lactone of ostrich oil was subjected to spectroscopic analysis (IR, NMR, and MS) and shown to be 5-hexyl-3H-furan-2-one (Figure 1). The lactone was isolated as a colorless oil with the molecular formula C_10_H_16_O_2_ as determined by EI-Ms [M]^+^: *m*/*z* = 168 (100%). The existence of a γ-lactone was suggested by IR carbonyl absorption at 1708 cm^−1^ (C=O) (Figure S3 in Supplementary File) and the IR spectrum of the sodium salt of the open form of the lactone (Figure S4 in Supplementary File) formed by the hydrolysis of the respective lactone, revealed absorption at 3426, 2922, 1563, 1448, and 868 cm^−1^. From which, absorption at 3426 cm^−1^ was assigned to the hydroxyl group and at 1563 and 1447 cm^−1^ for the carboxylate anion. The sodium salt of the open lactone was reverted back to the lactone when treated with dilute hydrochloric acid. ^1^H-NMR spectrum showed (CDCl_3_, 400 MHz, (Figure S1 in Supplementary File)) characteristic proton signals at δ H 0.9 (t, 3H, CH_3_, *J* = 7.0 Hz), 1.31–1.41 (m, 6H, 3 × CH_3_), 1.61 (quintet, 2H, *J* = 7.5 Hz), 2.31 (m, 2H), 3.21 (m, 2H, lactone CH_2_), 5.23 (m, 1H, alkene-H). While ^13^C-NMR spectrum displayed (CDCl_3_, 100 MHz, (Figure S2 in Supplementary File)) carbon signals at δ C 14.2 (CH_3_), 22.7, 25.7, 27.9, 28.9, 31.5 34.1 (6 CH_2_), 98.0 (alkene CH), 157.4 (alkene quaternary), 178.1 (C=O). Based on the interpretation of the above-mentioned data, it was proposed that the structure of the isolated compound was assigned to be the lactone (Figure 1). 5-hexyl-3H-furan-2-one has been isolated for the first time from ostrich oil as was previously synthesized as a building block in enantioselective Bronsted acid catalyzed *N*-acyliminium cyclization cascade reactions [16].

### 2.2. Nanoparticles Preparations

**Figure 2:**
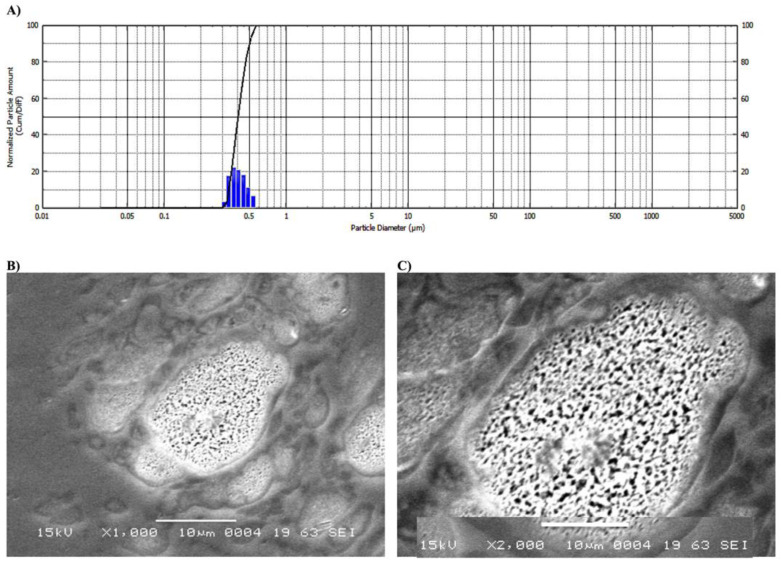
Characterization of the formulated γ-lactone-MC-NPs: (**A**) Particle size distribution, (**B**) Images taken with SEM at ×1000, and (**C**) Images taken with SEM at ×2000.

The size distribution for the prepared nanoparticles showed normal distribution with size diameter of 250 ± 13 nm (Figure 2A). γ-lactone-MC-NPs were formed in a stable nano-emulsion solution without any visible coalescence. Collectively, these results indicate a significant difference from **γ**-lactone itself which was completely immiscible in water. Nevertheless, the formulated γ-lactone-MC-NPs are completely dispersed in water (Figure 1). The entrapment of **γ**-lactone was confirmed by the change in color of MC to that of **γ**-lactone. Also, the size of **γ**-lactone-MC-NPs was increased to 250 ± 13 nm; this is attributed to the uniformity of γ-lactone-MC-NPs which showed only one peak without any aggregation. The particles were considered stable without aggregation after purification, and the formulated particles were also found to be relatively stable in size and did not form aggregates. The obtained data is considered as an intensity-weighted value and sensitive process for formulated smaller forms. This may be essential for aggregated or adulterated models [17,18,19].

The γ-lactone-MC-NPs had a spherical shape and a particle size of 13–14 μm as shown by SEM. γ-lactone-MC-NPs showed highly spotted with more uniform and non-aggregated particles (Figure 2, image B). This result also confirmed the dynamic light scattering (DLS) study findings. Moreover, the γ-lactone-MC-NPs showed spotted dot inside the vesicle which are attributed to the **γ-l**actone itself (Figure 2; images C). The γ-lactone-MC-NPs showed difference in size as the particle diameters from DLS were lower than those obtained from SEM imaging and this was attributed to the presence of the layer coating of the γ-lactone-MC-NPs which further limits the total particle density [20]. The sizes of the γ-lactone-MC-NPs recorded by the DLS and SEM showed difference owing to different techniques. Our interpretation agrees with the size recorded with DLS and SEM. The data from DLS showed the average sizes of γ-lactone-MC-NPs varied significantly from that measured by the SEM technique. The DLS based average size estimations of the γ-lactone-MC-NPs were also varied with times and may not be reproducible wherein the nano-emulsion may aggregate and affect the average distribution of size [20,21].

### 2.3. Fourier-Transform Infrared Spectroscopy (FTIR)

**Figure 3:**
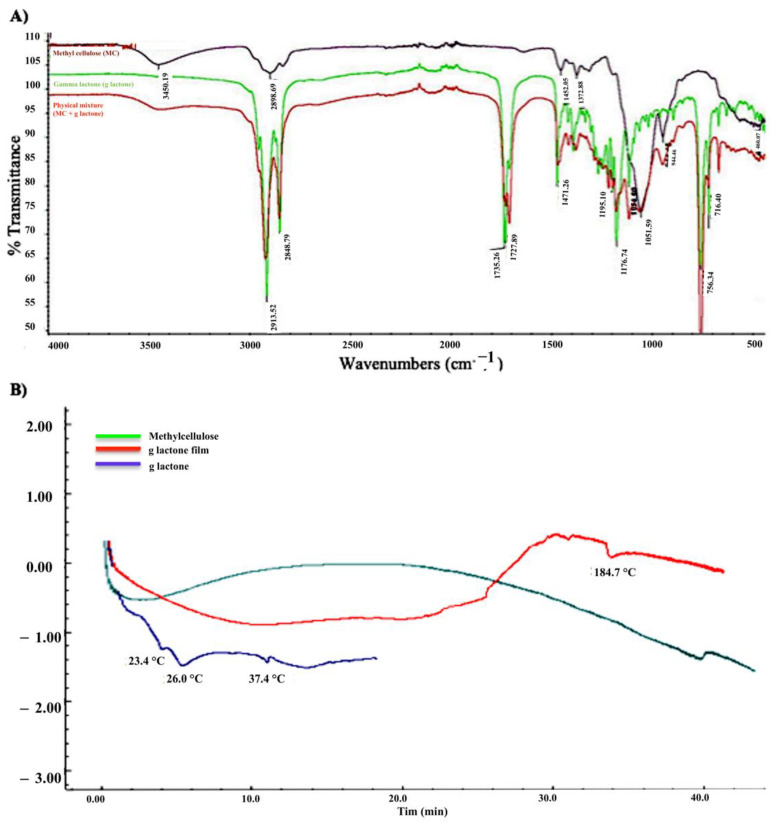
(**A**) FTIR spectra of γ-lactone (g lactone), methyl cellulose (MC), and γ-lactone in physical mixture with MC. The FTIR showed the typical peaks of γ-lactone without any foreign peaks due formulation. (**B**) Differential scanning calorimetry (DSC) for γ-lactone, MC, and γ-lactone in physical mixture with MC. Crosslinking of MC was detected forming plastic like film.

The functional groups of γ-lactone, MC, and γ-lactone in a physical mixture with MC were checked by FTIR (Figure 3A) to examine the drug interaction between γ-lactone and MC. Absorption bands showed no prominent interaction between the two compounds in the physical mixture which had similar absorption bands as their raw powders. Moreover, the FTIR studies showed no interaction between the γ-lactone and MC, which is consistent with other results discussed previously that used FTIR to confirm the physical interaction of all components but found no interaction between γ-lactone and MC which means the γ-lactone can be released from the formulated γ-lactone-MC-NPs [22,23].

### 2.4. Differential Scanning Calorimetry (DSC)

As shown in the (Figure 3B) γ-lactone exhibits three endothermic peaks, at 23.4, 26, and 37.4 °C. The physical crosslinking of methylcellulose (MC) is a consequence of the formation of hydrophobic domains. Crosslinking kinetics of MC and its final stiffness depends on different parameters of the used product, such as the substitution degree or molecular weight, but it also might be adjusted using various concentrations and additives. Crosslinking of MC was observed after complete drying of the nanoparticle’s formulation used in this study by forming plastic like film. The thermogram for the prepared nanoparticles showed a broad endothermic peak due to incorporation of γ-lactone into MC followed by an exothermic peak that indicates the formation of the crosslinking form of MC. A third endothermic peak at 184.7 °C was observed due to the melting of crosslinking MC [24].

### 2.5. Encapsulation Efficiency of γ-Lactone in Nanoparticles

The **γ**-lactone was incorporated in MC as γ-lactone-MC-NPs at 59.9%. This might be attributed to the low solubility of **γ-l**actone in water. Also, the presence of MC enhanced the entrapping of **γ**-lactone which was employed as an emulsion stabilizer in the distilled water (DW) emulsion.

### 2.6. Anti-Inflammatory Activity

In inflammations, cyclooxygenase (COX) is the key enzyme in the synthesis of autacoids [25]. Steroidal and non-steroidal anti-inflammatory drugs are currently the most widely used drugs in the treatment of acute inflammatory disorders, despite their renal and gastric negative secondary effects. As the result of long-term use of these drugs, the adverse effects become imminent such as gastric lesions and cardiovascular and renal failure [26]. Now, there is a need for new, safe, potent, and less toxic drugs. This stimulates our present study.

Previous studies on the ‘oil’ obtained from emu fat showed a very effective inhibition of chronic inflammation in rats when applied dermally (with a skin penetration enhancer) [27]. From our preliminary studies on saponification of ostrich oil, we noticed that the fatty acids obtained from ostrich oil showed similar composition to that of birds and rabbits and none of them showed anti-inflammatory property which is only rebutted for ostrich oil. The fatty acid profile showed: oleic acid (43.17%), palmitic acid (23.21%), and linoleic acid (16.88%), together with other fatty acids, in trace amounts: fatty acid, palmitoleic acid, linolenic acid, and lauric acid. Studies on the saponifiable fraction using chromatographic techniques furnished three compounds—A, B, and C. Compounds A and B from mass spectroscopy were shown to be high molecular weight hydrocarbons with simple functional groups, i.e., hydroxyl and ketone groups, respectively. Compound C showed to be the gamma lactone, which was focused on as the part which is responsible for the anti-inflammatory effect of ostrich oil. In light with similar results from the study on Emu oil, which showed the bulk of the anti-inflammatory activity, was present in the low triglyceride fraction [12]. This part of the fractionated oil was the concern of our present study.

**Figure 4:**
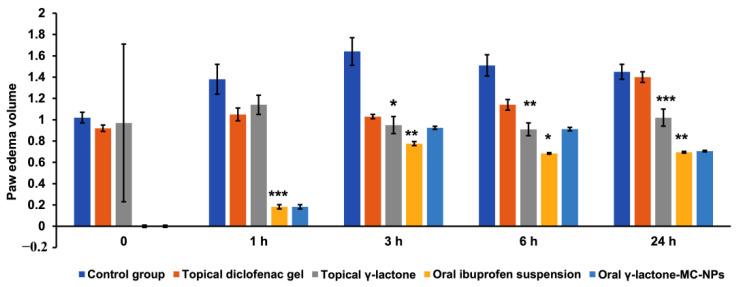
The percent of edema inhibition in cu.mm in the control group, topical diclofenac gel treated group, oral ibuprofen treated group, topical γ-lactone treated groups, and γ-lactone-MC-NPs treated group. Diclofenac gel and γ-lactone were administered at 300 mg diclofenac of 1% *w*/*w* and 30 mg γ-lactone. Ibuprofen suspension administered at 30 mg/kg, γ-lactone-MC-NPs was administrated at 10 mL/kg of γ-lactone (each 1 mL contains 223.5 μg). The results are expressed by Mean ± SEM (*n* = 30). One-way ANOVA followed by Tukey–Kramer multiple comparison test showed * *p* ˂ 0.05 ** *p* ˂ 0.01 and *** *p* ˂ 0.00 as compared to the positive control group and the reference group.

The topical application of γ-lactone, 5-hexyl-3H-furan-2-one, isolated from ostrich oil showed reduction of formalin induced paw edema starting from the first hour and continued up to 24 h. At the first hour the reduction effect produced by γ-lactone was mild as the mean paw was 1.14 ± 0.09 cu.mm compared to 1.05 ± 0.06 cu.mm in diclofenac gel treated and 1.38 ± 0.14 cu.mm in positive control and the percent inhibition of edema was 56.8% while in diclofenac gel it was 67.8% (Table 1 and Figure 4).

After 3 h, γ-lactone induced significant reduction in paw edema resulting in mean paw volume of 0.95 ± 0.08 cu.mm compared to 1.03 ± 0.02 cu.mm in diclofenac gel and 1.64 ± 0.13 cu.mm in the positive control group with *p-*value ˂ 0.05 indicating significant difference between groups. The percent inhibition of edema was also increased for both γ-lactone treated (79.3%) and diclofenac gel treated (74.6%). This study noticed that at three hours the edema reduction effect of γ-lactone started to increase and yield a greater effect than the reference diclofenac gel (Table 1 and Figure 4).

After 6 h, γ-lactone continued to show more anti-inflammatory activity than the reference diclofenac gel as the mean paw volume was 0.91 ± 0.06 cu.mm compared to 1.14 ± 0.05 cu.mm in diclofenac gel (*p-*value ˂ 0.01) and the anti-inflammatory effect was clearer in the percent inhibition of edema: 95.7% for γ-lactone treated and 75.2% for diclofenac gel treated (Table 1 and Figure 4).

After 24 h the diclofenac gel effect was nearly to be abolished as the mean paw volume was 1.40 ± 0.05 cu.mm approaching that of the positive control (1.45 ± 0.07 cu.mm), in contrast γ-lactone continued to show the anti-inflammatory effect even after 24 h as the mean paw volume was 1.02 ± 0.08 cu.mm (*p-*value ˂ 0.001). These results confirmed that γ-lactone has prolonged anti-inflammatory effect that continued beyond 24 h with percent inhibition of edema volume of 75.6% (Table 1 and Figure 4).

The oral administration of γ-lactone-MC-NPs to the rats at the first hour showed potent paw edema inhibition of 92.5% compared to ibuprofen oral emulsion at 81.8%. Oral γ-lactone-MC-NPs continued to show effective reduction of paw edema at the third and sixth hour, while after 24 h the percent of edema inhibition was almost the same as that produced by ibuprofen (Table 1 and Figure 4).

These results showed a significant inhibition of edema in both topical γ-lactone, and oral γ-lactone-MC-NPs with a higher effect in the later (*p-*value ˂ 0.001). The topical application of the γ-lactone and γ-lactone-MC-NPs were proved to be better than the reference drugs—diclofenac gel and ibuprofen emulsion. The anti-inflammatory activity lasts for up to 24 h as compared to the reference drugs. This result suggests a relatively potent and long-lasting effect for the topical γ-lactone and oral γ-lactone-MC-NPs over the reference drugs. The isolated γ-lactone; 5-hexyl-3H-furan-2-one is responsible for the anti-inflammatory activity of the ostrich oil.

### 2.7. The Histological Evaluation of Skin Tissues for Topical γ-Lactone

As shown in Table 2 and the raw data available in the Table S1 in Supplementary File, the histological assessment of skin thickness in a γ-lactone treated rats was compared to skin thickness in negative control, positive control and diclofenac treated groups. The results were expressed as mean ± SEM (*n* = 24). One-way ANOVA followed by the Tukey–Kramer multiple comparison test was performed and the comparison between groups was significant as the *p*-value is 0.00002 and the result was considered significant at *p* < 0.05. The comparison between the negative control and the positive control showed significant difference (*p*-value = 0.00003). The comparison between the negative control and the diclofenac treated rats was also significant (*p*-value = 0.023). While, the comparison between the negative control and the γ-lactone treated rats showed no difference as the *p*-value was insignificant (*p*-value = 0.899). The difference between the positive control and diclofenac treated and that between the positive control and γ-lactone treated were significant as the *p*-value was 0.0352 and 0.00013, respectively. However, there was insignificant difference between the diclofenac treated and γ-lactone treated rats, as the *p*-value was 0.096.

**Figure 5:**
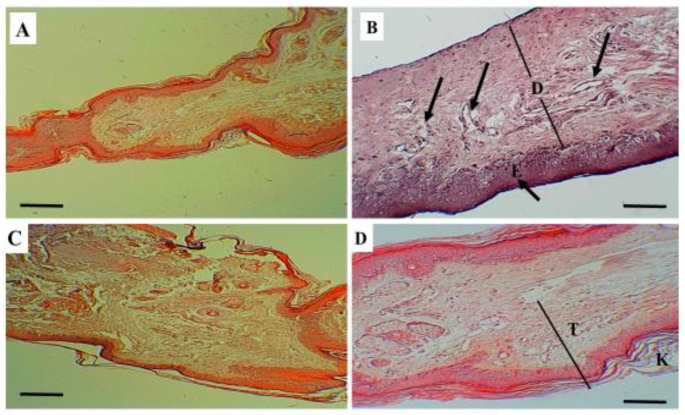
The histopathological assessment of skin thickness in rats: Photo (**A**): negative control, (**B**): Positive control, (**C**): Diclofenac treated, and (**D**): γ-lactone treated. E: epidermis (short arrow), D between two lines: Dermis, long arrow: loose tissue and dilated blood vessels, K: Keratinous layer, T: total thickness in lactone treated group. Scale bar: 100 μm. H&E satin.

The skin in positive control group showed sub epidermal edema with tortious dilated blood vessels, which lead to increase thickness of the skin. While in diclofenac treated and γ-lactone treated groups the skin showed significant reduction in thickness as compared to the positive control (Figure 5).

**Figure 6:**
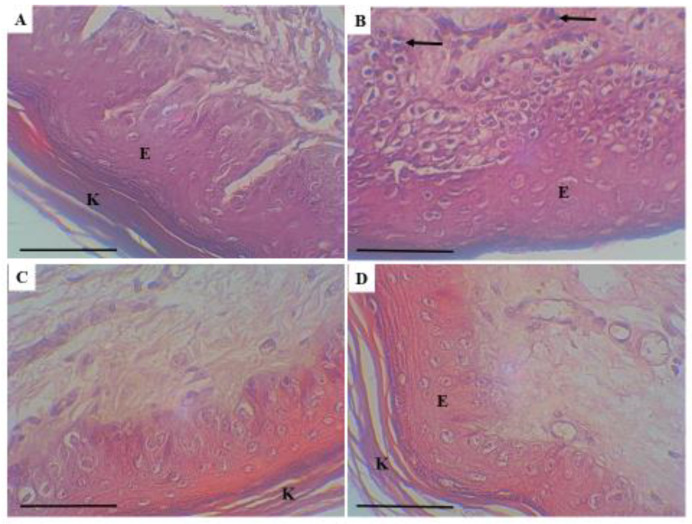
Histopathological assessment of inflammation in the skin of rats. Photo (**A**): negative control, (**B**): Positive control, (**C**): Diclofenac treated, and (**D**): γ-lactone treated. E: epidermis, arrows: inflammatory cells infiltration, K: Keratinous layer. Scale bar: 100 μm. H&E stain.

The skin of positive control also showed mixed inflammatory cellular infiltration, which was more intense in the dermal layer with scattered inflammatory cells in the base of the epidermis. Figure 6.

Histopathological evaluation showed a quantitative increase in the thickness of the skin in the positive control group as a result of edema and tortious dilatation of the blood vessels that developed after formalin injection. This increase in the thickness was statistically significant when compared to the negative control. The effect of formalin in tissue is well documented in previous studies [28,29].

This study also showed statistically significant anti-inflammatory effect of a γ-lactone compared to positive control and yielding a similar result to the diclofenac gel a recognized and widely used anti-inflammatory drug [30]. Moreover, the histopathological assessment revealed mixed infiltration of inflammatory cells, which was more prominent in the dermal layer and the basal part of the epidermis. The degree of inflammation was scored as intense inflammatory cellular infiltration (>40% of inflammatory cells in the sections) in the positive control group. Inversely, the degree of inflammation was markedly reduced and it was scored as slight inflammatory cellular infiltration (<20% of inflammatory cells in the sections) in both γ-lactone treated and diclofenac gel treated groups, which supports the hypothesis of anti-inflammatory effect of the γ-lactone against formalin-induced injury in rats.

### 2.8. Acute Toxicity and Weight Changes Study

From Table 3, the oral administration of γ-lactone-MC-NPs in a single dose of 5000 mg/kg at 20 mL/kg showed no toxic effect on the behavioral responses in the treated rats except for lethargy, drowsiness, and refusal of feeding observed in the first 4 h after dosing. There were no visual signs in skin, eyes, salivation, nor diarrhea in rats. After 24 h, no mortality occurred but significant weight loss (*p*-value 0.02 and 0.041) was observed. Apparently, the reason might be related to the refusal of feeding. After four days, 33.3% mortality in each group occurred. Although oral administration of the γ-lactone-MC-NPs showed potency in clearing paw edema in rats but lethargy, drowsiness, and refusal of feeding may limit its oral administration.

### 2.9. The Diuretic Effect of Oral γ-Lactone-MC-NPs

The parameters in Table 4 were calculated according to the formulae stated in the methods to compare the diuretic effects of oral γ-lactone-MC-NPs with a control and a standard diuretic. The diuretic action of oral γ-lactone-MC-NPs was found to be 1.01 which is similar to that of the control. Moreover, the diuretic activity of the oral γ-lactone-MC-NPs showed similar result to that of the control at 0.57 and 0.55, respectively. Diuretic activity in both the control and oral γ-lactone-MC-NPs apparently showed about 50% of the standard furosemide drug.

## 3. Materials and Methods

### 3.1. Extraction

The oil was obtained from traditional medicine shops in Sudan as a pale yellow oil that was saponified using a standard saponification procedure [31]. In which, 5 g of the oil was refluxed with 10% alcoholic KOH solution on water bath for 30 min. The alcohol was evaporated under vacuum, the residue was diluted with water, taken into a separatory funnel and extracted by dichloromethane to remove the higher hydrocarbons and then the aqueous layer was extracted with ethyl acetate. The ethyl acetate was evaporated under vacuum to yield semi-solid oil (3.2 g). The residue was fractionated through dry silica column using ethyl acetate as an eluent to give colorless semi-solid (2.9 g) as a major product. The residue was subjected to further separation as a result of the observation in a preliminary work that the anti-inflammatory activity was located in this extract.

### 3.2. Separation and Characterization

The crude ethyl acetate residue (500 mg) was purified using preparative thin layer chromatography using cyclohexane/ethyl acetate 60:40 as a developing solvent to yield a major compound (350 mg) as a low melting colorless solid. This was shown to be a lactone, from the ease of ring opening with 10% sodium hydroxide solution to give the sodium salt of hydroxy acid as a pale yellow solid, m.p. 245 °C. The lactone ring closed back on reaction with dilute hydrochloric acid, (λ max 1708 cm^−1^ for the lactone carbonyl stretch and at 3426 cm^−1^ for the hydroxyl group of the sodium salt). Further spectroscopic analyses showed this to be a γ-lactone, Figure 1. ^1^H-NMR spectrum showed (CDCl_3_, 400 MHz) characteristic proton signals at δ H 0.9 (t, 3H, CH_3_, *J* = 7.0 Hz), 1.31–1.41 (m, 6H, 3 × CH_3_), 1.61 (quintet, 2H, *J* = 7.5 Hz), 2.31 (m, 2H), 3.21 (m, 2H, lactone CH_2_), 5.23 (m, 1H, alkene-H). While ^13^C-NMR spectrum displayed (CDCl_3_, 100 MHz) carbon signals at δ C 14.2 (CH_3_), 22.7, 25.7, 27.9, 28.9, 31.5 34.1 (6 CH_2_), 98.0 (alkene CH), 157.4 (alkene quaternary), 178.1 (C=O). Molecular formula C_10_H_16_O_2_ [M]^+^: *m*/*z* = 168 (100%). The γ-lactone was the subject of the anti-inflammatory activity in the present study.

### 3.3. Nanosuspension Preparation of γ-Lactone

γ-lactone was embedded in soluble nanoparticles (NPs) to increase the solubility of γ-lactone. These nanoparticles were made using MC polymer as the outer coating layer. γ-lactone-MC-NPs were prepared as the method stated previously with some modifications [32,33]. Briefly, 1 g of MC was dissolved in 100 mL distilled water and stirred continuously until a clear solution was obtained (Stock solution I). Further, 50 mg of γ-lactone were dissolved in 10 mL ethyl acetate (stock solution II). Moreover, dispersions containing 0.5 wt.% γ-lactone with respect to 1 g MC were prepared with 10 mL of ethyl acetate water and the dispersions were sonicated to obtain a uniform dispersion without agglomerates (Stock solution I and II). Both γ-lactone and MC mixture were mixed together and stirred continuously to let the ethyl acetate evaporate and a clear solution without bubbles was obtained and then left to stir for 12 h. The obtained nanoparticles were then characterized accordingly.

### 3.4. Size Measurements

Shimadzu particle size analyzer (SLAD-400, Shimadzu, Kyoto, Japan) was used to measure the size of γ-lactone-MC-NPs and the weight average of 10 of microparticles was announced. The samples were adjusted to 25 °C and exposed to a 633-nm laser beam at a 90° scattering angle. All samples have been conducted in an aqueous solution [34].

### 3.5. Determination of Surface Morphology

A volume of 20 µL of γ-lactone-MC-NPs solution were placed on the surface double-sided copper conductive tape and were let to dry. The γ-lactone-MC-NPs were then coated with a thin layer of platinum in a vacuum chamber (Zeny Vacuum pump, CA, USA) for 55 s at 25 mÅ, γ-lactone-MC-NPs solution were then sputter-coated (using Sputter coater, JOEL JFC-1300, Peabody, MA, USA) to create γ-lactone-MC-NPs electrically conductive before imaging in SEM. Both morphology and size of γ-lactone-MC-NPs were investigated using JEOL JSM-550 Scanning Electron Microscope (SEM) (Jeol, Akishima, Tokyo, Japan) [35].

### 3.6. Fourier Transform Infrared (FTIR) Spectroscopy

Fourier transform infrared (FTIR) γ-lactone, MC, and γ-lactone-MC physical mixture was performed using (Bruker, OPTIK GmbH, Type: Tensor 27, Ettlingen, Germany). The samples were scanned at the range of 400–4000 cm^−1^. For purification, the γ-lactone-MC-NPs was centrifuged at 12500 rpm, for 20 min, and were washed with Millipore water three times [8].

### 3.7. Differential Scanning Calorimetry (DSC)

The thermal profiles of all materials and mixtures used in this study were measured by DSC-60 (Shimadzu, Kyoto, Japan) using 4–6 mg of sample in open aluminum pans, with an empty pan as a reference. The temperature increased with a heating rate of 20 °C/min for **γ**-lactone from 18 to 60 °C and from 18 to 220 °C for pure MC as well as for the final nanoparticle formulation prepared from the mixture of MC and **γ**-lactone (*w*/*w*) under a nitrogen gas flow.

### 3.8. Determination of Entrapment Efficiency %

The entrapment efficiency percent (EE%) of **γ**-lactone in **γ**-lactone-MC-NPs was determined spectrophotometrically. A volume of 10 mL of nanosuspension was mixed with 10 mL ethyl acetate, the mixture was stirred for 1 h until **γ**-lactone-MC-NPs dissolved and **γ**-lactone was completely released. The solution was measured for encapsulation efficiency of **γ**-lactone using a UV spectrophotometer (Varian Cary^®^ 50 UV-Vis Spectrophotometer, Port Melbourne, Australia) at a wavelength of 388 nm. Drug content was computed using a calibration curve (R^2^ = 0.9998) prepared from **γ-l**actone solutions with a concentration of 1–6 µg/mL. The EE% of **γ-l**actone was calculated according to the following equation:

(1) $$\mathrm{EE}\%=\frac{\left(Total\;amount\;of\;drug\;in\;particle\right)}{\;weight\;of\;particles\;taken}\times 100$$

### 3.9. Animal Study

The study protocol was approved by the Ethics Committee for Animal Care and Use in the College of Pharmacy-Qassim University (Approval ID 2019-CP-9) [36].

#### 3.9.1. Anti-Inflammatory Evaluation

Thirty male albino rats that were 12 to 16 weeks old, weighing 116–198 g, were obtained from the animal house, College of Pharmacy, Qassim University, Saudi Arabia and kept in standard conditions. The animals were divided randomly into five groups of six each.

#### 3.9.2. Formalin-Induced Edema in the Left Paw of Rats

Edema was induced in the left hind paw of rats in all groups with sub planter injection of 0.1 mL of 5% formalin as described previously [36]. The right hind paw was maintained as a negative control. The anti-inflammatory activity was compared with control and treated groups using the plethysmograph method [36,37]. To all groups of rats, a mark at the tibia–tarsal junction was made with a permanent marker on both hind paws to ensure constant paw volume. In all groups, the left and right paw volume was measured at zero time (normal paw volume) and at 1, 3, 6, and 24 h after induction of inflammation.

The percent inhibition of edema was calculated as follow:

(2) $$\%\;\mathrm{Edema}\;\mathrm{inhibition}\;=\frac{\left(VL-VR\right)control-\left(VL-VR\right)treated}{\left(VL-VR\right)control}\times 100$$

where *VL* represents the mean of the left paw displacement volume and *VR* represents the mean of the right paw displacement volume.

#### 3.9.3. Application of Diclofenac Gel and γ-Lactone

One group received 300 mg of 1% *w*/*w* diclofenac gel by applying it topically on the left hind paw 10 min before induction of inflammation [38]. Another group received the 30 mg of the neat γ-lactone isolated from the ethyl acetate extract of ostrich oil by applying it topically on the left hind paw 10 min before induction of inflammation. The last group was regarded as the positive and negative controls—R (Right paw)-negative control, L (Left paw) positive control.

#### 3.9.4. Oral of Ibuprofen Suspension and γ-Lactone-MC-NPs

For the oral administration, ibuprofen was used as reference and medicated as γ-lactone-MC-NPs were orally administered at a volume of 5 mL/kg from one day at 0, 1, 3, 6, and 24 h. Ibuprofen suspension was administered at single daily doses of 30 mg/kg which was given orally 10 min before induction of the inflammation. The γ-lactone-MC-NPs was administrated at 10 mL/kg of γ-lactone (each 1 mL contains 223.5 μg) isolated from ostrich oil as a base nano-emulsion was given orally 10 min before induction of inflammation. γ-lactone and ibuprofen suspension, respectively, were administered 0.02 mL of 5% formalin by intra plantar injection in the left hind paw [39].

#### 3.9.5. Acute Toxicity and Weight Changes Study

Acute oral toxicity was performed according to the Organization of Economic Cooperation and Development guidelines 420 for testing chemical compounds. Nine rats of both sexes aged 8 to 16 weeks-old were categorized in to groups 1, 2, and a control group. All rats were fasted for 18 h. γ-lactone-MC-NPs was administered as an oral gavage in a single dose of 5000 mg/kg at 20 mL/kg to rats in group 1 and 2 (*n* = 6) while deionized water was administered similarly to the control group (*n* = 3). All animals were allowed free-access to water before and after treatments but food was provided after 2 h of treatment. Observation like mortality, physical behavior, feeding, and other signs were carried out for four days.

#### 3.9.6. Diuretic Activity of Oral γ-Lactone-MC-NPs

Diuretic activity was determined following the methods used by Lahlou et al. The Diuretic action and diuretic activity were calculated according to the Formulae (1) and (2), respectively.

$$1.\;\mathrm{Diuretic}\;\mathrm{action}=\frac{Urinary\;excretion\;of\;treatment\;group}{Urinary\;excretion\;of\;Control\;group}\;2.\;\mathrm{Diuretic}\;\mathrm{activity}=\frac{Diuretic\;action\;of\;test\;drug}{Diuretic\;action\;of\;standard\;drug}$$

### 3.10. Histological Evaluation of Tissue

Three days after induction of inflammation, both right and left hind paws’ slits were fixed in 10% formalin and left for 24 h for fixation. The formalin fixed tissue was further processed using automated tissue processor machine (Leica TP1020, Leica Microsystems GmbH, Wetzlar, Germany) and paraffin imbedded sections were prepared. Serial 3-µm sections were prepared using microtome (Leica RM2245, Leica Microsystems GmbH, Wetzlar, Germany) and stained by hematoxylin and eosin stain. All tissue sections were examined by light microscope (Olympus BX41, Olympus, Tokyo, Japan) using 4×, 10×, 20×, and 40× magnifications and the image was taken via a digital image camera (5MP Binocular Microscope Electronic Eyepiece USB Video CMOS Camera for Image Capture, Zhejiang ULIRVISION Technology Co., Ltd, Zhejiang, China) and Top View image analyzer for more accurate measurement. The skin thickness quantification was measured in millimeters and the mean was derived from assessing eighteen sections form each group. Qualitative assessment of vascular tortuosity and dilatation together with edematous changes was performed. The inflammatory cellular infiltration was assessed in eighteen sections from each group and it was scored in to slight (presence of <20% of inflammatory cells in the section), moderate (presence of 20–40% of inflammatory cells in the section), and intense (presence of >40% of inflammatory cells in the section) as described in a previous study [40,41].

### 3.11. Statistical Analysis of Results

The volume measured by plethysmometer test and the thickness of the dermis were expressed as mean ± Standard Error of Mean. Paired *t*-test and One-Way ANOVA test followed by Tukey–Kramer multiple comparison tests were used to compare the mean between different groups and the result was considered significant at *p-*value < 0.05.

## 4. Conclusions

The present study showed that the γ-lactone, 5-hexyl-3H-furan-2-one, isolated from ostrich oil significantly lowers the paw edema in injured rats, which proved to be superior to the effect of diclofenac gel and ibuprofen suspension. The histological examination showed a significant reduction in skin thickness and inflammation. This study proved that the γ-lactone is solely responsible for the anti-inflammatory effect of ostrich oil, which agrees with a similar study on emu bird that showed this effect. These findings support its folkloric use.

## Figures and Tables

**Table 1:** The anti-inflammatory activity of the γ-lactone-1 compared to that of the reference diclofenac gel expressed as percent inhibition of edema (*n* = 30) (Raw data available in the Tables S2–S4 in Supplementary File).

Test	Anti-Inflammatory Activity and Percentage Inhibition of Edema (Mean)				
	0 h	1 h	3 h	6 h	24 h
Control group	0	0	0	0	0
Diclofenac gel treated *	0%	67.8%	74.6%	75.2%	26.2%
Oral Ibuprofen suspension **	0%	81.8%	72.3%	71.98%	77.5%
γ-lactone treated *	0%	56.8%	79.3%	95.7%	75.6%
Oral γ-lactone-MC-NPs ***	0%	92.5%	88.3%	89.76%	77.9%

* Diclofenac gel and γ-lactone were administered at 300 mg diclofenac of 1% *w*/*w* and 30 mg γ-lactone. ** Ibuprofen suspension administered at 30 mg/kg; *** γ-lactone-MC-NPs was administrated at 10 mL/kg of γ-lactone (1 mL contains 223.5 μg).

**Table 2:** The histological changes in skin-thickness measured in (mm) (*n* = 24). Diclofenac gel and γ-lactone were applied at 300 mg diclofenac of 1% *w*/*w* and 30 mg γ-lactone.

Number	Negative Control (Right Paw)	Positive Control (Left Paw)	Diclofenac Gel Treated	γ-Lactone Treated
1	1.0054	1.3361	0.9075	1.0451
2	0.9895	1.2779	1.1536	0.8281
3	0.8916	1.3758	1.2859	0.9789
4	0.7699	1.1906	1.0716	1.0477
5	0.9524	1.1986	1.1800	0.9842
6	0.8281	1.2912	0.9895	0.8043
Mean ± SEM	0.9062 ± 0.038	1.2784 ± 0.030	1.098 ± 0.056	0.9481 ± 0.036

**Table 3:** Acute toxicity and weight changes study.

Groups	Weight of Rat in Grams in 0 h (Mean ± SD)	Weight of Rat in Grams in First 24 h (Mean ± SD)	*p*-Value	Initial Observation up to 4 h	Weight of Rat at Day 4
Control group (*n* = 3)	232.7 ± 13	232.7 ± 13	-	Normal	229.4 ± 11.2
Group 1 (*n* = 3)	168.7 ± 11.5	160.7 ± 11.4	0.02 *	Drowsiness, Sedation, Lethargic, and refusal of feeding	165.1 ± 12.6 One dead (33.3%)
Group 2 (*n* = 3)	261.0 ± 13.0	250 ± 13.0	0.041*	Drowsiness, Sedation, Lethargic, and refusal of feeding	258.9 ± 10.8 One dead (33.3%)

* *p*-value < 0.05 is considered significant. Oral γ-lactone-MC-NPs administered in a single dose of 5000 mg/kg at 20 mL/kg.

**Table 4:** The diuretic effect of oral γ-lactone-MC-NPs in rats.

Volume of Urine (mL) Mean ± SD							
Group	1st Hour	2nd Hour	3rd Hour	4th Hour	5th Hour	Diuretic Action	Diuretic Activity
Control (*n* = 3)	0.61 ± 0.015	0.84 ± 0.025	0.93 ± 0.010	1.01 ± 0.015	1.05 ± 0.020	1	0.55
Furosemide (*n* = 3)	1.16 ± 0.03	1.44 ± 0.030	1.63 ± 0.030	1.77 ± 0.025	2.03 ± 0.015	1.81	1
γ-lactone-MC-NPs (*n* = 3)	0.64 ± 0.015	0.81 ± 0.006	0.95 ± 0.010	1.04 ± 0.006	1.03 ± 0.020	1.01	0.57

## Data Availability

Data were provided in the text and supplementary file.

## Supplementary Materials

Figure S1: H-NMR spectrum of compound **1**; Figure S2: C-NMR spectrum of compound **1**; Figure S3: FT-IR spectrum of compound **1**; Figure S4: FT-IR spectra of the Na^+^-salt of compound **1**; Table S1: The histological changes in skin—thickness measured in (mm).; Table S2: The anti-inflammatory activity result of control group in Plethysmometer test.; Table S3: The ant-inflammatory activity result of 30 mg/kg of oral ibuprofen suspension in Plethysmometer test.; Table S4: The anti-inflammatory activity result of 10 mL/kg of The γ-lactone nano emulsion base in Plethysmometer test.

## Sample Availability

Sample of compound 1 is available from the first author.
