# Supplementary material for: The Anti-Inflammatory Effect of a γ-Lactone Isolated from Ostrich Oil of Struthio camelus (Ratite) and Its Formulated Nano-Emulsion in Formalin-Induced Paw Edema
Source: Molecules. 2021 Jun 17;26(12):3701. doi: 10.3390/molecules26123701 (PMC8234909; doi:10.3390/molecules26123701)
Supplement: Supplementary file 1 [file molecules-26-03701-s001.zip › molecules-1226770-supplementary.pdf]

molecules

Supplementary Material

The Anti-Inflammatory Effect of a γ-Lactone Isolated from Ostrich Oil of *Struthio camelus* (Ratite) and Its Formulated Nano-Emulsion in Formalin-Induced Paw Edema

Salah E. M. Eltom [1,*], Ahmed A. H. Abdellatif [2,3], Hamzah Maswadeh [2], Mohsen S. Al-Omar [1], Atef A. Abdel-Hafez [1], Hamdoon A. Mohammed [1,4,*], Eiman ME. Agabein [5], Ibrahim Alqasoomi [2], Salem A. Alrashidi [1], Mohammed S. M. Sajid [6] and Mugahid A. Mobark [7,8]

1 Department of Medicinal Chemistry and Pharmacognosy, College of Pharmacy, Qassim University, Buraydah 51452, Saudi Arabia; m.omar@qu.edu.sa (M.S.A.-O.); a.abdelgalil@qu.edu.sa (A.A.A.-H.); salem11098@gmail.com (S.A.A.)
2 Department of Pharmaceutics, College of Pharmacy, Qassim University, Buraydah 51452, Saudi Arabia; A.Abdellatif@qu.edu.sa (A.A.H.A.); msodh@qu.edu.sa (H.M.); imq3911@gmail.com (I.A.)
3 Department of Pharmaceutics and Industrial Pharmacy, Faculty of Pharmacy, Al-Azhar University, Assiut 71524, Egypt
4 Department of Pharmacognosy, Faculty of Pharmacy, Al-Azhar University, Cairo 11371, Egypt
5 Department of Histology, College of Medicine, Qassim University, Buraydah 51452, Saudi Arabia; eimanagabein@qumed.edu.sa
6 Department of Pharmacology and Toxicology, College of Pharmacy, Qassim University, Qassim 51452, Saudi Arabia; su.mohammed@qu.edu.sa
7 Department of Pharmacy Practice, College of Pharmacy, Qassim University, Buraydah 51452, Saudi Arabia; mu.mohammed@qu.edu.sa
8 Department of Pathology, Faculty of Medicine and Health Sciences, University of Kordofan, El-Obied 157, Sudan
* Correspondence: S.Eltom@qu.edu.sa (S.E.M.E.); ham.mohammed@qu.edu.sa (H.A.M.)

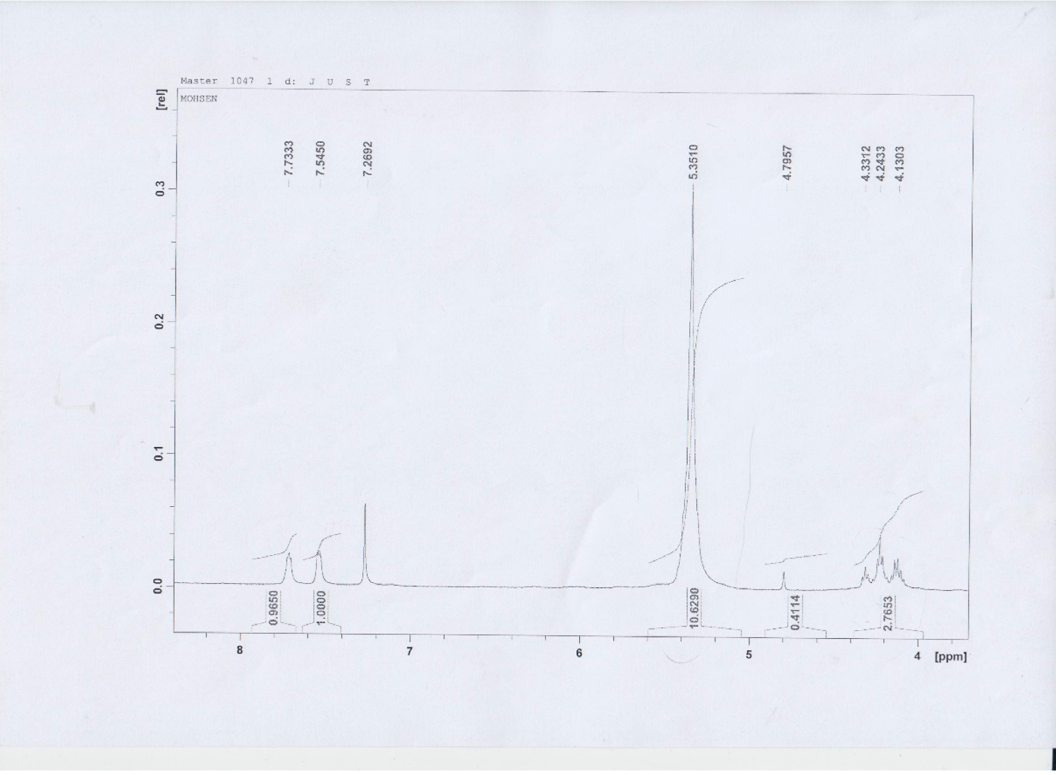

Figure S1. ^{1}H-NMR spectrum of compound **1**.

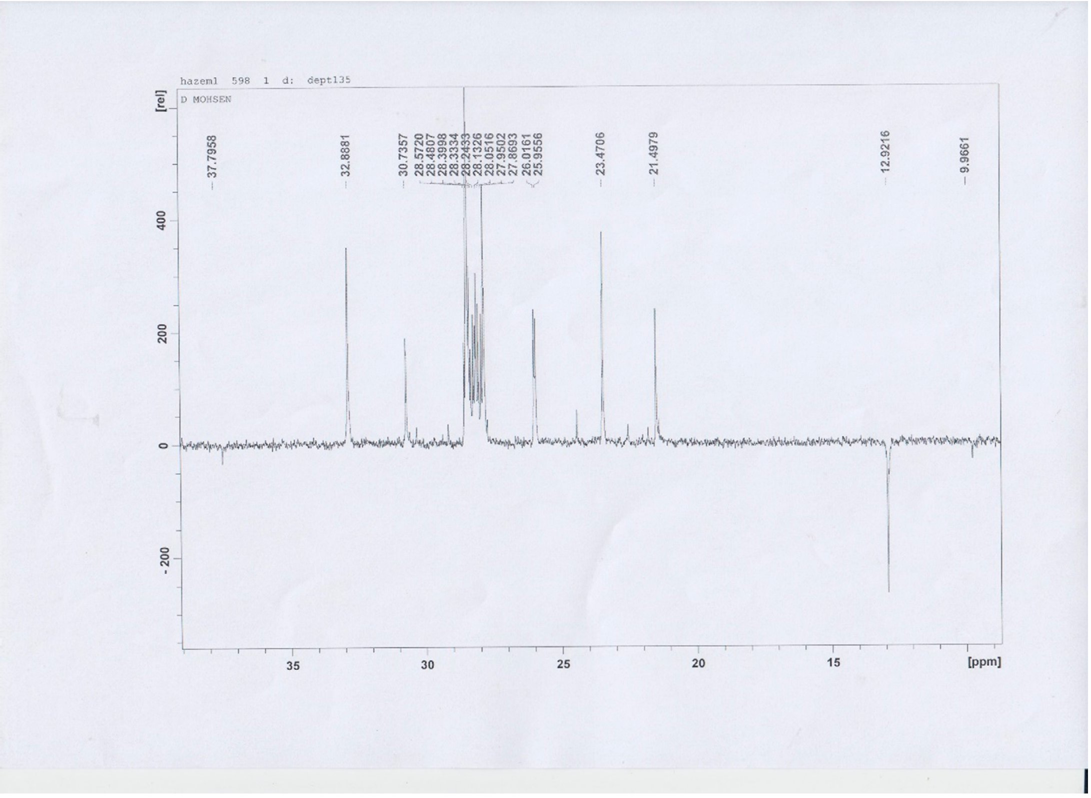

Figure S2. ^{13}C-NMR spectrum of compound **1**.

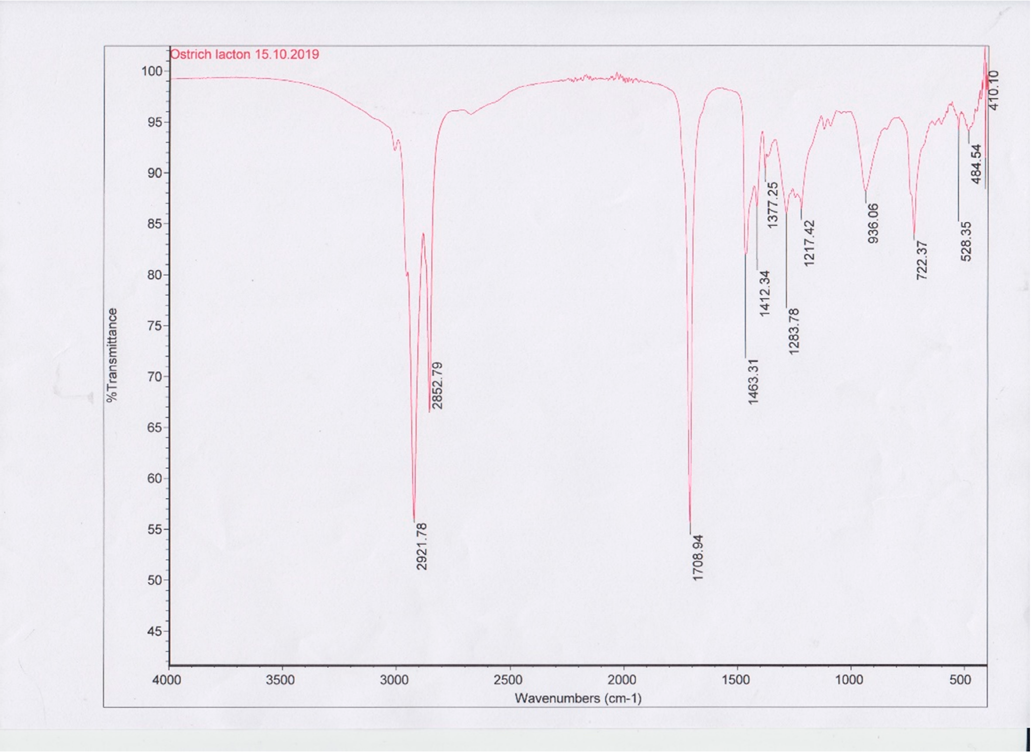

Figure S3. FT-IR spectrum of compound **1**.

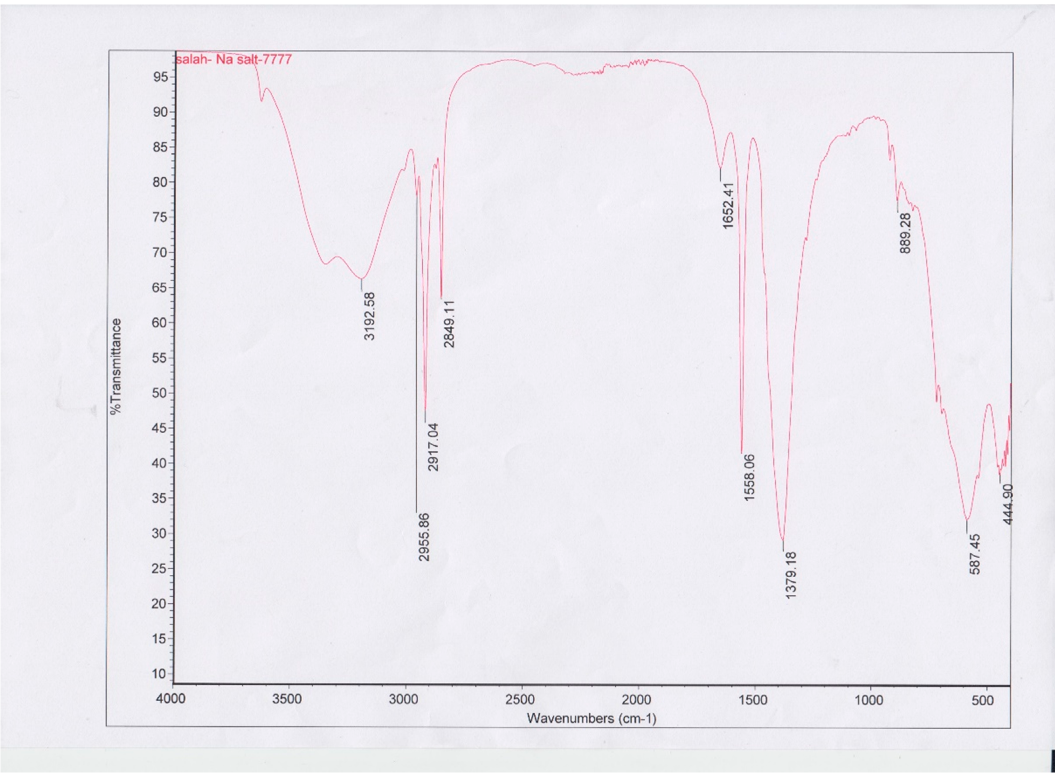

Figure S4. FT-IR spectra of the Na⁺-salt of compound **1**.

Table S1. The histological changes in skin - thickness measured in (mm).

**Negative Control (Right Paw)**				
**Number**	**Section 1**	**Section 2**	**Section 3**	**Average**
1	1.0043	1.0061	1.0059	1.0054
2	0.9897	0.9896	0.9891	0.9895
3	0.8915	0.8913	0.8919	0.8916
4	0.7699	0.7701	0.7696	0.7699
5	0.9522	0.9528	0.9523	0.9524
6	0.828	0.8279	0.8285	0.8281
Mean ± SEM				0.9062 ± 0.038
Positive Control (Left Paw)				
Number	Section 1	Section 2	Section 3	Average
1	1.3363	1.3362	1.3358	1.3361
2	1.278	1.2779	1.2777	1.2779
3	1.3755	1.3759	1.376	1.3758
4	1.1907	1.1902	1.1909	1.1906
5	1.1986	1.1984	1.1989	1.1986
6	1.2909	1.2914	1.2913	1.2912
Mean ± SEM				1.2784 ± 0.030
Diclofenac Gel Treated				
Number	Section 1	Section 2	Section 3	Average
1	0.9071	0.9077	0.9076	0.9075
2	1.1537	1.1536	1.1534	1.1536
3	1.2854	1.2861	1.2863	1.2859
4	1.0718	1.0709	1.072	1.0716
5	1.1792	1.1803	1.1805	1.18
6	0.9892	0.9893	0.9899	0.9895

Mean ± SEM				1.098 ± 0.056
Diclofenac Gel Treated				
Number	Section 1	Section 2	Section 3	Average
1	1.0453	1.0449	1.0452	1.0451
2	0.8283	0.828	0.8279	0.8281
3	0.9789	0.9791	0.9787	0.9789
4	1.048	1.0475	1.0477	1.0477
5	0.9844	0.9843	0.9838	0.9842
6	0.8044	0.8043	0.8043	0.8043
Mean ± SEM				0.9481± 0.036

The histological changes in skin - thickness measured in (mm)
Derived for the above tables

Number	Negative Control (right paw)	Positive Control (left paw)	Diclofenac Gel Treated	γ- Lactone Treated
1	1.0054	1.3361	0.9075	1.0451
2	0.9895	1.2779	1.1536	0.8281
3	0.8916	1.3758	1.2859	0.9789
4	0.7699	1.1906	1.0716	1.0477
5	0.9524	1.1986	1.1800	0.9842
6	0.8281	1.2912	0.9895	0.8043
Mean ± SEM	0.9062 ± 0.038	1.2784 ± 0.030	1.098 ± 0.056	0.9481± 0.036

Table S2. The anti-inflammatory activity result of control group in Plethysmometer test.

**S.no**	**Body Weight (G)**	**Paw Volume (mL) as Measured by Mercury Displacement at Time.**									
		0 h		1 h		3 h		6 h		24 h	
		R	L	R	L	R	L	R	L	R	L
1	28	0.19	0.21	0.17	0.29	0.17	0.36	0.20	0.39	0.21	0.35
2	34	0.20	0.21	0.19	0.28	0.18	0.32	0.18	0.45	0.20	0.39
3	18	0.12	0.11	0.14	0.19	0.15	0.33	0.13	0.37	0.12	0.31
4	24	0.14	0.14	0.14	0.26	0.15	0.35	0.14	0.34	0.13	0.30
5	26	0.20	0.22	0.13	0.37	0.15	0.44	0.15	0.48	0.15	0.41
6	32	0.16	0.16	0.16	0.24	0.16	0.39	0.15	0.40	0.15	0.33
Mean ± SEM	27 ± 2.35	0.17 ± 0.01	0.175 ± 0.009	0.155 ± 0.009	0.27 ± 0.02	0.16 ± 0.005	0.37 ± 0.01	0.16 ± 0.01	0.41 ± 0.02	0.16 ± 0.01	0.35 ± 0.01

Table S3. The ant-inflammatory activity result of 30mg/kg of oral ibuprofen suspension in Plethysmometer test.

**S.no**	**Body weight (G)**	**Paw Volume (mL) as Measured by Mercury Displacement at Time.**									
		0		1 h		3 h		6 h		24 h	
		R	L	R	L	R	L	R	L	R	L
1	26	0.23	0.20	0.23	0.31	0.20	0.25	0.22	0.29	0.20	0.25
2	24	0.18	0.18	0.19	0.28	0.20	0.21	0.18	0.29	0.22	0.25
3	38	0.27	0.25	0.28	0.38	0.28	0.23	0.25	0.30	0.27	0.30
4	30	0.20	0.21	0.21	0.33	0.19	0.33	0.20	0.29	0.20	0.25
5	30	0.19	0.20	0.19	0.26	0.19	0.21	0.20	0.27	0.16	0.25
6	24	0.16	0.17	0.15	0.25	0.17	0.28	0.17	0.25	0.15	0.25

Mean ± SEM	28.667 ± 2.171	0.205 ± 0.016	0.202 ± 0.011	0.208 ± 0.018	0.302 ± 0.020	0.205 ± 0.016	0.252 ± 0.019	0.203 ± 0.012	0.282 ± 0.007	0.200 ± 0.018	0.258 ± 0.008

Table S4. The anti-inflammatory activity result of 10ml/kg of The γ-lactone nano emulsion base in Plethysmometer test.

**S.no**	**Body Weight (G)**	**Paw Volume (mL) as Measured by Mercury Displacement at Time.**									
		0		1 hr		3 hr		6 hr		24 hr	
		R	L	R	L	R	L	R	L	R	L
1	14	0.17	0.16	0.15	0.25	0.15	0.17	0.17	0.18	0.23	0.24
2	14	0.17	0.19	0.18	0.30	0.18	0.18	0.18	0.16	0.21	0.22
3	20	0.24	0.21	0.22	0.35	0.23	0.20	0.23	0.25	0.25	0.28
4	20	0.18	0.21	0.18	0.39	0.19	0.23	0.19	0.25	0.24	0.23
5	26	0.20	0.23	0.22	0.35	0.22	0.27	0.22	0.25	0.24	0.22
6	24	0.19	0.20	0.20	0.33	0.18	0.20	0.17	0.20	0.19	0.22
Mean ± SEM	19.667 ± 2.028	0.192 ± 0.011	0.200 ± 0.010	0.192 ± 0.011	0.328 ± 0.020	0.192 ± 0.012	0.208 ± 0.015	0.193 ± 0.011	0.215 ± 0.016	0.198 ± 0.016	0.254 ± 0.007
